# Evaluation of the dietary intake data coding process in a clinical setting: Implications for research practice

**DOI:** 10.1371/journal.pone.0221047

**Published:** 2019-08-12

**Authors:** Vivienne X. Guan, Yasmine C. Probst, Elizabeth P. Neale, Linda C. Tapsell

**Affiliations:** 1 School of Medicine, Faculty of Science, Medicine and Health, University of Wollongong, Wollongong, New South Wales, Australia; 2 Illawarra Health and Medical Research Institute, University of Wollongong, Wollongong, New South Wales, Australia; Tabriz University of Medical Sciences, ISLAMIC REPUBLIC OF IRAN

## Abstract

**Background:**

High quality dietary intake data is required to support evidence of diet-disease relationships exposed in clinical research. Source data verification may be a useful quality assurance method in this setting. The present pilot study aimed to apply source data verification to evaluate the quality of the data coding process for dietary intake in a clinical trial and to explore potential barriers to data quality in this setting.

**Methods:**

Using a sample of 20 cases from a clinical trial, source data verification was conducted between three sets of data derived documents: transcripts of audio-recorded diet history interviews, matched paper-based diet history forms and outputs from nutrition analysis software. The number of cases and rates of discrepancies between documents were calculated. A total of five in-depth interviews with dietitians collecting and coding dietary data were thematically analysed.

**Results:**

Some 2024 discrepancies were identified. The highest discrepancy rate was 57.49%, and occurred between diet history interviews and nutrition analysis software outputs. Sources of the discrepancies included both quantities and frequencies of food intake. The highest discrepancy rate was for the food group “vegetable products and dishes”. In-depth interviews implicated recall bias of trial participants as a cause of discrepancies, but dietitians also acknowledged a possible subconscious influence of having to code reported foods into nutrition analysis software programs.

**Conclusion:**

The accuracy of dietary intake data appeared to depend on the level of detailed food data required. More support for participants on reporting consumption, and incorporating supportive tools to guide estimates of food quantities may facilitate a more consistent coding process and improve data quality. This pilot study offers a novel method and an overview of dietary intake data coding measurement errors. These findings may warrant further investigation in a larger sample.

## Introduction

The highest level of evidence for nutrition policy and clinical practice comes from randomised controlled trials (RCTs). Food-based RCTs aim to make changes in dietary intake during interventions and test the effects on health outcomes. As dietary intake is an important behavioural risk factor that can be targeted to improve health [1], the quality of evidence from related RCTs is important and collecting accurate dietary intake data is contributes to this quality. However, generating dietary intake data accurately remains a challenge. Although biomarkers can provide accurate estimations of intake over a defined time period, limited recovery biomarkers are known to reflect energy and nutrient intakes (e.g., protein, sodium and potassium) [2, 3]. Food intake data is required to translate diet-disease relationships into practical recommendations [4], and self-report dietary assessment tools are applied to derive food-based dietary intake data [5].

It is recommended that self-report dietary assessment methods should be validated, ideally prior to the study initiation [6], allowing for measurement errors in the dietary assessment tool to be identified. This can assist in reducing and correcting measurement errors and improving the quality of data. For example, a standardized dietary assessment method (data derivation process) can be implemented, addressing potential measurement errors and allowing for calibration using linear regression [6]. Rather than the process of data collection, these validation studies tend to focus on the outcome, dietary intake data, addressing relative validity. The validation studies provide evidence on different degrees of validity for different dietary components of the tested dietary assessment tool, and when administered it in particular populations and settings [7]. Examining validity from the perspective of the process of dietary intake data derivation is another matter.

In clinical research settings, open-ended dietary assessment methods such as food records and diet history interviews tend to be used to generate dietary intake data [8]. The data is generated by a stepwise process including collection and coding [9]. Importantly, coding dietary data into a database is not a simple process, particularly for data derived from open-ended methods. A wide range of foods can be described with different levels of detail by study participants during data collection. In practice, the data coding process involves coding the food items, and the intakes of quantities (portion sizes) and/or frequencies into the available nutrition analysis software platform to reflect the reported dietary intake. If the reported food item or portion size cannot be found in the software, commercial and cultural food knowledge, as well as professional judgment are required to find an appropriate substitute [10, 11]. The literature suggests that experienced coders also face challenges in making subjective decisions on matched items in the database [12]. Thus, data coding is complicated by how dietary data is captured. Literature suggests that designing data coding processes largely relies on a professionals’ experience [10, 13], or consideration of food choices seen in population surveys [14]. Moreover, the major component of dietary intake data quality in nutrition appears to be the evaluation of coded dietary intake data [15–20]. Thus, in this research we consider the issue of the data collection process to identify factors that may influence the quality of dietary data coding, and thereby data quality in the clinical research setting.

The International Conference on Harmonisation for Good Clinical Practice provides a standard for the quality of clinical trial data, and suggests applying on-site monitoring and/or central monitoring to make sure that the data is accurate and complete [21]. Given source data is the principal information collected in a clinical trial, source data verification (SDV) has been used primarily to ensure the quality of data. SDV is the process of ensuring that the data used for analyses are accurately transcribed from the original source data documents [22]. Although growing evidence reveals that performing SDV to evaluate data appears to have a negligible effect on data quality [23], the complexity of current clinical trials may play a critical role in challenging the traditional method, such as SDV. Advanced operational practices are required to manage such complexity [24]. Thus, quality management methods, such as source data review has been proposed to provide strategic quality management beyond data [25]. Although performing SDV is time-consuming, laborious and costly [26], given the nature and complexity of the dietary intake data coding process, it may offer detailed information about dietary coding discrepancies such as the types, trends and the data points related to the coding process in a given dataset. The findings from performing SDV may thus contribute to improvement of dietary intake data quality. In addition, the literature suggests that data quality assessment should apply mixed methods including qualitative (e.g., interview and reviewing documentation) and quantitative assessment methods (e.g., an audit of data) with an assessment of multiple sources of data (e.g., records, data collection process and documentation) [27]. Using mixed methods not only objectively measures data quality, but also assists in describing and understanding the issues influencing data quality in depth to develop effective strategies for data quality improvement [28]. This pilot study aimed to evaluate the dietary intake data coding process in a food-based intervention RCT and to investigate barriers to accurate coding of dietary intake data.

## Methods

The present pilot study applied a mixed methods approach, using SDV and in-depth interviews with dietary intake data collectors and coders. To investigate the dietary data coding process, SDVs were used to compare information obtained from transcripts of audio-recorded diet history interviews, matched paper-based diet history records and outputs from a nutrition analysis software program. To put this in context, in-depth interviews with Accredited Practising Dietitians (APDs) who collected and coded the dietary intake data was conducted aimed at exploring the challenges with the dietary intake data coding practice.

### Study participants and recruitment process

The present study was approved by the University of Wollongong/Illawarra Shoalhaven Local Health District Human Research Ethics Committee (HE15/014). Participants were volunteers in the food-based RCT (referred to as trial participants) and dietitians, who collected and coded dietary intake data (referred to as trial dietitians). The food-based RCT is registered at the Australian and New Zealand Clinical Trial Registry (ANZCTRN 12614000581662). Prior to data collection, study information and consent forms were distributed to and collected from trial participants by email and trial dietitians by face-to-face. The literature suggests that 10% of trial participants in a sample are required for the SDV analysis to explore data quality [15–19, 29]. Data were only collected on those participants willing to participate in the quality audit.

**Fig 1:**
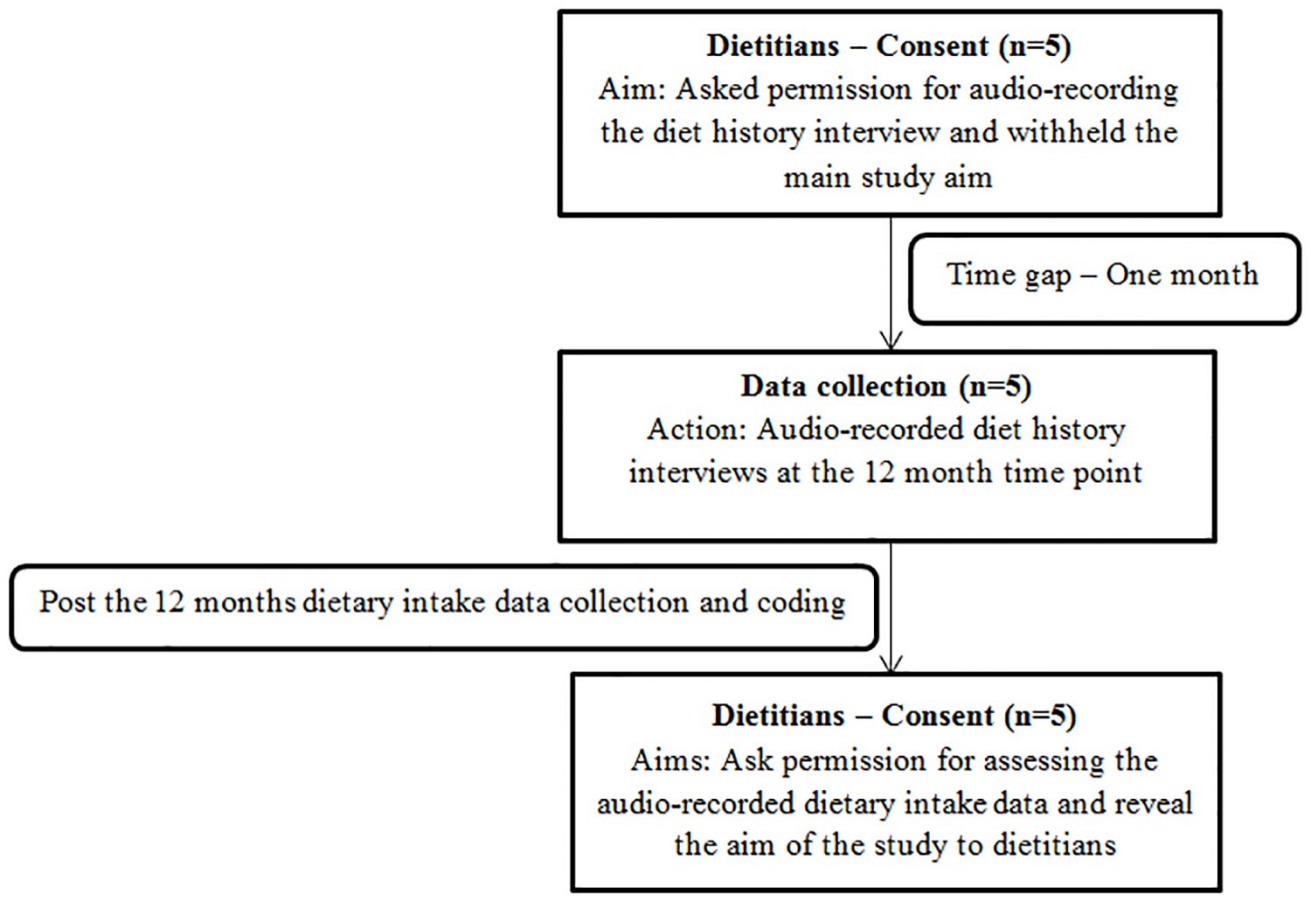
Participant flow of obtaining study consent from the trial dietitians in the food-based trial.

The process of obtaining study consent from the trial dietitians is presented in Fig 1. During distributing the forms to the trial dietitians requesting permission to audio-record diet history interviews, the trial dietitians were made aware that they would be audio-recorded, the main purpose was withheld. The rationale behind withholding the aim until the end of the study period was to avoid behaviour changes in the presence of an audio recorder (Hawthorne effect) [30, 31]. A one-month time gap was created between the consent form distribution and the audio recording to decrease the trial dietitians’ awareness of the recording process [32]. This ensured that the trial participants and trial dietitians were not aware of which diet history interviews were being recorded. The digital audio-recorders were placed in the consultation room and were hidden. An example of the audio-recorder locations in the consultation room is shown in S1 Fig. The allocations of diet history interviews between the trial dietitians and the trial participants were determined by dietitian and participant availability, which was independent of the present study. As all trial dietitians who were involved with the duration of the study were interviewed, data saturation was attained for this part of the study.

### Diet history interview and dietary intake data coding process

The present analysis was based on the raw dietary intake data of the food-based RCT at the 12-month time point as a sub-sample of cases [33, 34]. The basis of this work was the diet history interviews of the trial, collected between July 2016 and April 2017. The diet history interviews and the dietary intake data coding were performed by APDs following the validated diet history interviews protocol using an open-ended face-to-face interview [35], who were blinded to the RCT study arms. During the interview, the trial participants were asked to recall their dietary intake on a usual day since the last assessment (typically over three months). The trial dietitians asked questions to clarify reported food items, the intake quantities and frequencies. Food models, measurement cups, utensils and plates were used to assist the trial participants to identify the portion sizes. A short food frequency checklist of omitted food items was employed by the trial dietitians [36].

The collected dietary intake data were coded to enable data entry to FoodWorks Professional nutrient analysis software (Xyris, QLD, Australia, Version 7, 2007). Food items were coded by selecting items from drop-down lists in the software supported by the AUSNUT 2007 food composition database [37]. Where appropriate, new foods were created by trial dietitians and added to the database to accurately reflect trial participant reported intakes. Food quantities were coded using a numeric assignment based on the standard units presented in the software to reflect the intakes of quantities. Intake frequencies were coded in the blank fields to indicate the intake variations.

Standard operating procedures (SOPs) for diet history interviews and the dietary intake data coding were developed prior to the RCT. These procedures included the introduction of the interviewer to trial participants, using portion estimation aids to assist trial participants to determine portion sizes, responsibility and requirements around the dietary intake data coding, and the process of recording assumptions made during the dietary intake data coding. Prior to the dietary intake data coding, all trial dietitians were trained using the SOPs including portion size conversion for the dietary intake data coding. The coded dietary intake data in Foodworks software was reviewed against paper-based records by a second APD to correct any outstanding errors.

### Source data verification

The voices of audio-recorded diet history interviews were digitally altered to de-identify the trial dietitians using Audacity software (ver. 2.1.1, available at http://audacity.sourceforge.net). The altered voices were checked by senior researchers (YP, EN) to ensure that the voices were unable to be identified. The diet history interviews were transcribed verbatim by author VG. The transcripts were reviewed against the original recordings by researchers who were independent of this study and the RCT.

The SDV process was performed by an APD (VG) independent of diet history interviews and the dietary intake data coding of the RCT. The matched paper-based diet history interviews case report forms (CRFs) and FoodWorks software output of food items and their quantities and frequencies, along with the transcripts of the audio-recorded diet history interviews were extracted. The data points of each document, including the transcripts of audio-recorded diet history interviews, CRFs and food outputs of FoodWorks, were the sum of the single food items and their quantities and frequency of intakes. For the transcripts of audio-recorded diet history interviews, the food items that the trial participants reported in the transcripts as consumed were counted. All the data points (100%) listed on the source data were verified manually against the CRFs or the FoodWorks software output.

**Fig 2:**
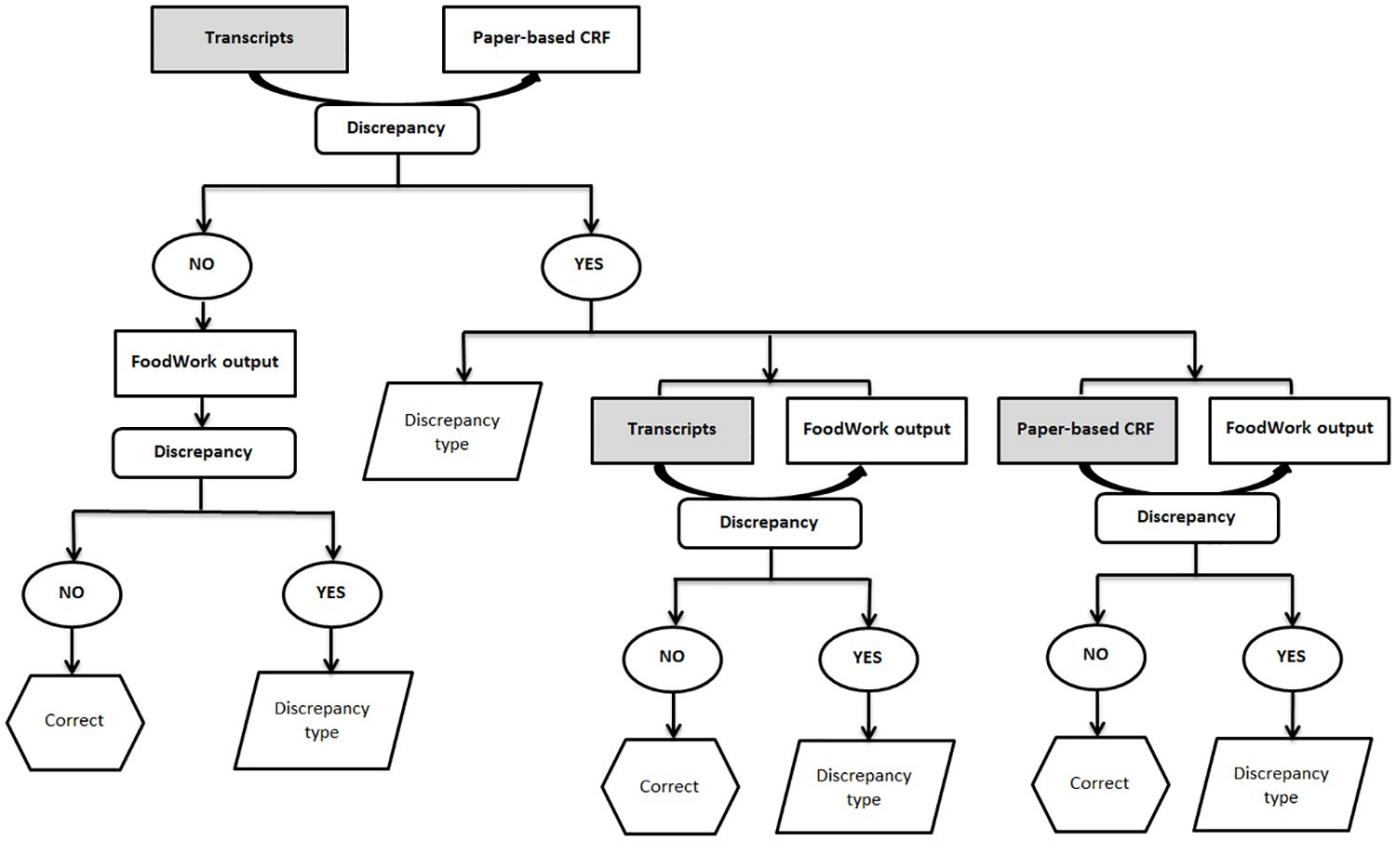
The source data verification flow of procedures^1, 2^.

There were three phases of verifications, indicating three paired document verifications. The detailed SDV process is shown in Fig 2. A dietary intake data discrepancy coding system was adapted from previous studies conducted with the same dataset (Table 1) [38, 39]. The AUSNUT 2007 major food groups were used to assess discrepancies about food groups [37]. The food codes and food group names of the AUSNUT 2007 are presented in S1 Table.

**Table 1:** Definitions and examples of discrepancy types^1^ [38, 39].

Discrepancy type	Definition	Example
Incorrect	Recorded on source document coded incorrectly or not related to items to the data destination	Recorded as two cups of bean stir fry and coded as one cup
Missed/missing	Recorded on source document but not coded to the data destination	Recorded two cups of bean stir fry but not coded to database
Sourceless	Not recorded on source data documents, but the data destination contains an entry	The quantity of bean stir fry not recorded on CRF, database record shows one cup
Questionable	Mismatch between source data documents and the data destination or detail of ingredients for a dish are listed on source data documents but pre-defined dish selected in the data destination	Recorded as bean stir fry in CRF, and transcribed as bean (mixed and canned) in the database

^1^CRF: case report form

Discrepancies identified from the transcripts were then re-coded in FoodWorks and outputs were compared with the original FoodWorks entries, and intakes of energy and macronutrients (protein, total fat, carbohydrate and fibre) were explored. Those discrepancies unable to be re-coded were retained in the software in their original form.

### Statistical analysis

The discrepancy rate was calculated by the total number of food items in source documents [17]. The discrepancy rate was calculated as:

$$\frac{\text{Total}\;\text{number}\;\text{of}\;\text{discrepancies}}{\text{Total}\;\text{number}\;\text{of}\;\text{food}\;\text{items}\;\text{in}\;\text{the}\;\text{source}\;\text{document}}\times 100$$

Discrepancy rates were calculated based on the number of food items from the source documents.

Statistical analyses were performed using the SPSS software package (SPSS version 21: IBM Corp. Released 2012. IBM SPSS Statistics for Windows, Version 21.0. Armonk, NY: IBM Corp). Normality of all data was checked using the Shapiro-Wilks test. Mean and standard deviation were presented for normally distributed data, and the median and interquartile range was reported for non-normally distributed data. Statistical significance was considered at p<0.05. One-way repeated measures ANOVA with Bonferroni correction was applied to assess the differences in the number of data points between the transcripts and CRFs, the transcripts and the food outputs of FoodWorks, and the CRFs and the food outputs of FoodWorks. The differences between daily intakes of energy and macronutrients in the original and re-coded data were explored using a paired t-test for parametric data, and the Wilcoxon signed rank test for non-parametric data; where the transcripts which could not be re-coded in FoodWorks were excluded from the analyses.

### In-depth interviews analysis

In-depth face-to-face interviews were conducted at the completion of the trial (August 2016 to September 2016) by a single APD (VG) independent of diet history interviews and the dietary intake data coding process in the workplace, following a semi-structured interview guide (S2 Table). The questions were designed based on the findings of a previous analysis of the dietary intake data coding process of the trial [38, 39] and were expanded to allow exploration of barriers to the dietary intake data generation process. The interview guide was assessed for face validity by senior researchers (YP, EN) prior to use.

The in-depth interviews were audio recorded and transcribed verbatim. Transcripts were reviewed against recordings by a researcher independent of this study (GW) and verified by the investigator (VG) to ensure accuracy. The “framework” approach proposed by Ritchie and Spencer [40] was used to guide the data analysis [41]. Coding occurred by reading all transcripts in full [42]. The categorization of the themes was agreed through the iterative process. Initial coding and thematic analysis to identify the dominant themes were conducted by the investigator (VG) and reviewed by senior researchers (YP, EN). Discrepancies were resolved through discussion until consensus was reached. Exemplar quotes for each theme were identified by the investigator (VG) and reviewed by senior researchers (YP, EN). The exemplar quotes supported each theme were reported. All themes were managed and reviewed using the qualitative analysis software QRS NVIVO, version 10.0 (QSR International Pty Ltd, Doncaster, VIC, Australia).

## Results

### Source data verification

From 178 participants who completed a diet history interview at 12 months, a total of 31 trial participants provided consent to participate in the present study, and 11% of the trial participant diet history interviews were analysed (n = 20). The reasons for exclusion were due to a change in the scheduled location and time (n = 7), technical issues in the audio-recorders which automatically stopped during the recording (n = 2) and being unable to place the audio-recorder due to another study running in the same consultation room (n = 2). The numbers of diet history interviews performed by the trial dietitians ranged from two to nine (n = 20). The characteristics of trial participants are presented in S3 Table.

The average length of audio-recorded diet history interviews was 27.47 ± 7.21 minutes. A total of 14,755 data points were verified from the transcripts, the CRFs and the food outputs of FoodWorks in the sub-sample. There was a significant difference in the total data points among three documents (p<0.0005). The number of data points of food items between three documents were not significantly different (p = 0.431, with Bonferroni correction), whereas the number of data points of intake of quantities and frequencies was significantly different among the three documents (p<0.0005 for intake of quantities with Bonferroni correction, p<0.0005 for intake of frequencies with Bonferroni correction). A summary of the number of data points and discrepancies between the transcripts, the CRFs and the food outputs of FoodWorks is shown in Table 2.

**Table 2:** Relevant number of data points, discrepancy type, number of discrepancies and discrepancy rate.

	Item	Quantity	Frequency	Total
**Number of data points per document**				
**Transcript**	88.15±31.77	67.05±24.37	75.15±29.21	230.35±80.46
**CRF**^1^	86.8±31.21	72.45±26.10	86.55±31.55	245.80±85.48
**Food output of FoodWorks**	87.2±30.78	87.2±30.78	87.20±30.78	261.60±92.33
**p value**^2^	p = 0.431	p<0.0005	p<0.0005	p<0.0005
**Discrepancy: Transcripts versus CRFs**^1^				
**Data points mean difference**^3^	1.35±0.72	-5.40±1.65	-11.40±2.79	-15.45±3.00
**Incorrect**	9 (10.59%)	24 (16.55%)	95 (27.70%)	128 (22.34%)
**Missed/missing**	47 (55.29%)	5 (3.45%)	5 (1.46%)	57 (9.95%)
**Sourceless**	20 (23.53%)	113 (77.93%)	233 (67.93%)	366 (63.87%)
**Questionable**	9 (10.59%)	3 (2.07%)	10 (2.92%)	22 (3.84%)
**Total number of discrepancies**	85 (4.82%)	145 (8.22%)	343 (19.46%)	573 (32.50%)
**Discrepancy: Transcripts versus FoodWorks**				
**Mean difference in number of data points**^3^	0.95±1.43	-20.15±4.26	-12.05±2.71	-31.25±6.14
**Incorrect**	23 (15.75%)	45 (9.51%)	106 (27.97%)	174 (17.43%)
**Missed/missing**	62 (42.47%)	12 (2.54%)	11 (2.90%)	85 (8.52%)
**Sourceless**	43 (29.45%)	415 (87.74%)	252 (66.49%)	710 (71.14%)
**Questionable**	18 (12.33%)	1 (0.21%)	10 (2.64%)	29 (2.91%)
**Total number of discrepancies**	146 (8.41%)	473 (27.25%)	379 (21.83%)	998 (57.49%)
**Discrepancy: CRFs**^1^ **versus FoodWorks**				
**Mean difference in number of data points**^3^	-0.40±1.12	-14.75±4.10	-0.65±0.83	-15.80±4.92
**Incorrect**	14 (22.58%)	24 (7.16%)	31 (55.36%)	69 (15.23%)
**Missed/missing**	15 (24.19%)	8 (2.39%)	6 (10.71%)	29 (6.40%)
**Sourceless**	23 (37.10%)	303 (90.45%)	19 (33.93%)	345 (79.16%)
**Questionable**	10 (16.13%)	0 (0%)	0 (0%)	10 (2.21%)
**Total number of discrepancies**	62 (3.56%)	335 (19.21%)	56 (3.21%)	453 (25.97%)

^1^CRFs: case report forms

^2^p values are for differences in the number of data points among the transcripts, CRFs and food output of FoodWorks in food items, quantities and frequencies.

^3^Mean ± Standard deviation

The total number of identified discrepancies was 2,024 (14.48% for food items, 47.08% for the intake of quantities, and 38.44% for the intake of frequencies). Nearly half of the discrepancies (49.31%) were identified from the verification between transcripts and the food outputs of FoodWorks. Using the transcripts as the source data, the discrepancy rates per CRF and food output were 11.87 ± 6.79% and 18.80 ± 10.69%, respectively. The discrepancy rate per food output was 8.15 ± 8.35% compared with source data from the CRF.

For food items, the most common discrepancy type was “missed/missing” in the three paired verification sets, whereas “sourceless” was the most common for intake quantities between the transcripts compared with the CRFs and the FoodWorks food output, respectively. The “incorrect” discrepancy type was the most common when CRFs and the food outputs of FoodWorks were compared.

**Fig 3:**
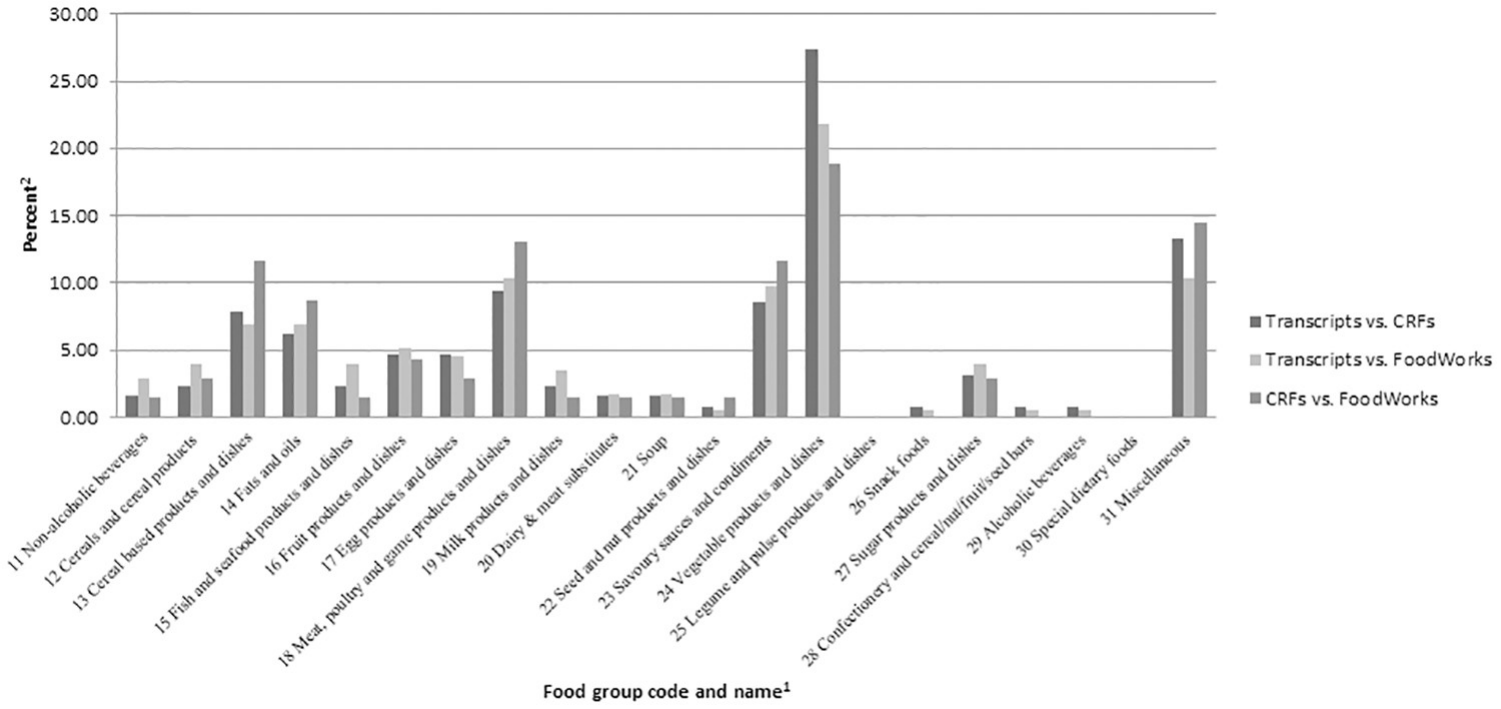
Percent of “incorrect” discrepancies identified in each food groups^2,3^.

The “vegetable products and dishes” food group presented the highest discrepancy rates in the three paired verification sets (32.46% for the transcripts vs the CRFs, 40.58% for the transcripts vs the food outputs of FoodWorks and 49.45% for the CRFs vs the food output of FoodWorks) (Fig 3). In the verifications between the transcripts and the CRFs, and between the transcripts and the food outputs of FoodWorks, the discrepancy type of “sourceless” of intake of quantities and frequencies was the major contributor of the discrepancy rates. The food groups with the “incorrect” discrepancy type are shown in Fig 3.

There were 17 cases which required re-coding from the transcripts into FoodWorks. All of the cases of “incorrect” were re-coded. Additionally, a total of ten missed/missing food items were re-coded, as their quantities and frequencies of intakes were also available. A total of 20.44% of discrepancies between the transcripts and food outputs of FoodWorks were re-coded from the transcripts to the FoodWorks. The median difference in energy intake between the original and re-coded data was 103.70 (interquartile range: -63.7–286.25) kJ/day. More than a 1MJ (1000 kJ/239 kcal) of daily energy intake difference between the original and re-coded data was identified for three transcripts. However, there was no significant difference between the original and re-coded data in intakes of daily energy (p = 0.136), protein (p = 0.198), total fat (p = 0.072), carbohydrate (p = 0.252) and fibre (p = 0.059).

### In-depth interview

**Fig 4:**
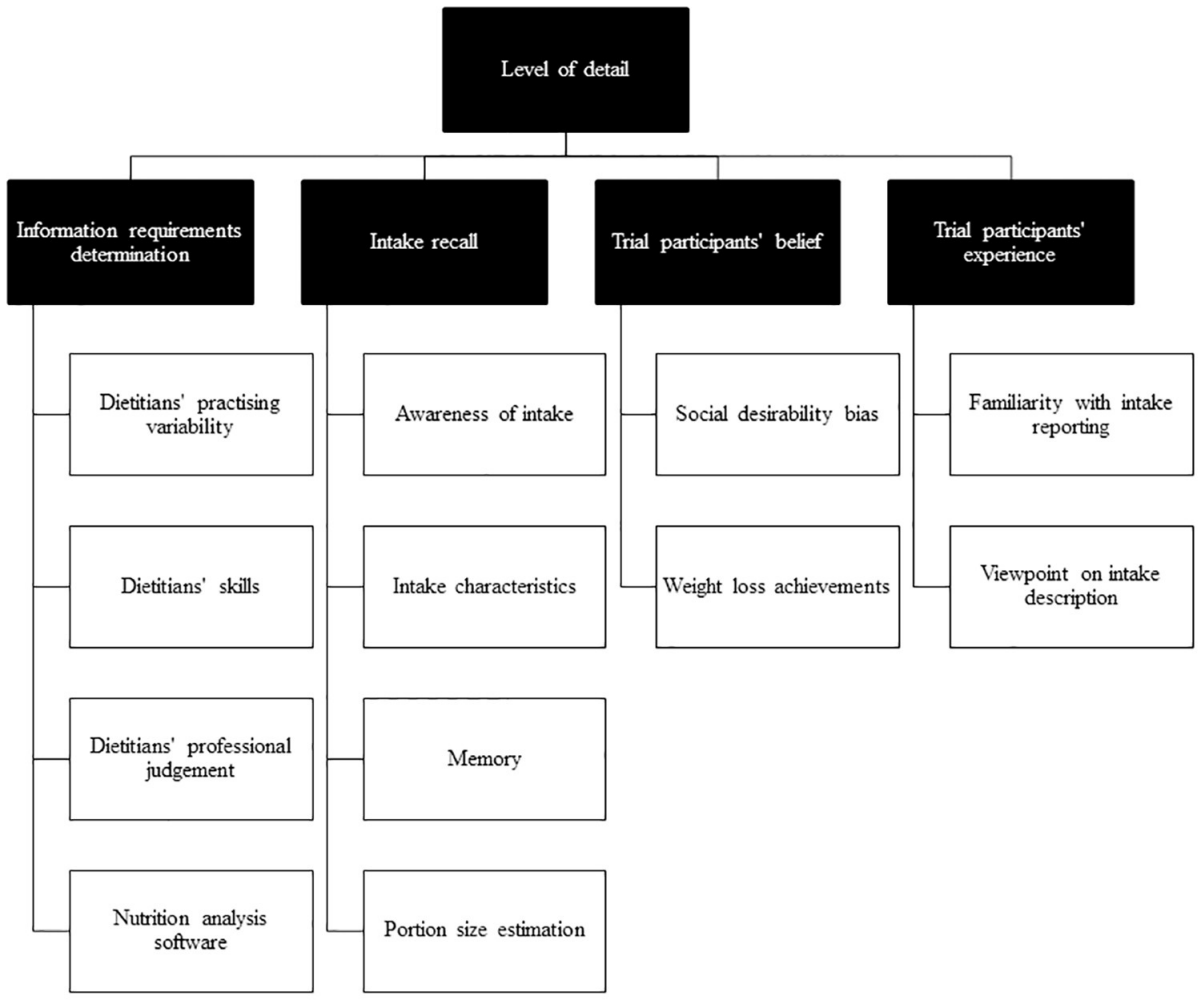
Identified themes affecting the quality of dietary intake data coding process under the main barrier *Level of detail*.

All the interviewed trial dietitians were female (n = 5). A total of three trial dietitians had worked as trial dietitians for three to five years, and two had worked nine to ten years. There was one trial dietitian who had previously worked in the community setting. The other four trial dietitians had worked in a research setting including three who had worked in private practice/consultancy. In-depth interviews lasted between 45 and 60 minutes. Analysis of interview data identified 17 dominant themes (Fig 4). The theme is italicised in the text. From the schematic analysis, the main driver of the quality of the dietary intake data coding process was the *level of detail* of dietary intake data. The trial dietitians agreed that dietary intake data in clinical trials required collecting adequate details for coding it into the nutrition analysis software, FoodWorks. Matching of food items in the nutrition analysis software relied on the *level of detail* of the food item description.

‘……There are so many brand names of foods, which might not all be in the FoodWorks …… they say they have chocolate every day or for certain time, and then you ask them which type of chocolate, and give the brand name, so you write it there, and then you come to check the brand of that chocolate, and Google it to see how it would look like, then of course then goes to ingredient list and, you know description, and then you look for the similar one, something else in the FoodWorks…’

(D2)

The *level of detail* of dietary intake data was reported to be dependent on the dominant themes including *dietitians’ information requirement determination*, *trial participants’ intake recall*, *trial participants’ belief of their intake* and *trial participants’ experience of data collection process*.

Firstly, trial dietitians’ decisions around the level of detail required for diet history interviews were determined by the level of detail required by the nutrition analysis software, as well as their professional judgment.

‘…… cause you want to get an idea of the overall intake, so you want them to at least give you, to have that full seven days, or that full month worth of foods, because when you put it into the FoodWorks, it needs to add up……’

(D5)

‘I think the other thing is, knowing we need to enter this data into FoodWorks, you try to tailor your questioning around the things, you know that FoodWorks is going to ask for as well’

(D3)

Coding the dietary intake data collected by other trial dietitians also revealed that the standards used for diet history interviews and trial dietitian’ diet history interview skills varied. Assumptions regarding intake were made by trial dietitians for information with an inadequate level of detail.

‘I usually base it [missed intake], so if I’m looking in a diet history, I either look at previous diet history from that same person, or I look at, so if something like meat for dinner, I look at all the other meats for dinner, and if that’s very similar amounts, I usually make the assumption to put that in, then I put a note in the FoodWorks that I made an assumption. Otherwise, I look at the diet history either side to look at whether they have the same recipe, and then if they have the same amount.’

(D4)

Trial dietitians reported that trial participants’ recall of intake was poor. Some trial participants did not pay attention to what they consumed, particularly those who did not cook their own meals. Given the current dietary assessment method aimed to recall usual dietary intake over the past three months, trial dietitians reported that trial participants easily recalled the meals of breakfast and lunch, but were unable to remember the dinner meal, which was likely due to the intake variations and mixed visible and invisible ingredients in a dish.

‘……If they [trial participants were] not involved in their meal preparation or shopping, they less aware of what they eat. So they are unable to give me an idea of um ye you know kind of food and drinks particularly with portion sizes as well’

(D1)

Despite their purpose to aid diet history interviews, memory aids to assess portion size appeared to be a barrier to accurately reporting quantities, particularly for loose foods (e.g., ready to eat breakfast cereal, rice and pasta); though food models were reported as the most useful tools.

‘……I often have a wide variety of cups and spoons out in front of them [trial participants], but they’ll often always goes to the smallest one, no matter what it is. So, and often using food models, sometimes that’s a barrier in terms of you can put the food models in front of them and say how much compared to that, but they just say the food model.’

(D4)

Furthermore, as the aim of the trial was weight loss, weight loss achievement also played a role in trial participants’ willingness to report their intakes and the level of detail of intake.

‘And especially those who are getting positive results…… if the goal was to lose, you know, a certain amount of weight, or to lose weight ……When they come the following if they lost a little bit weight,……They are motivated and feel oh this is going well, so when you start to talking foods, asking what they eat, they are ready to, you know, give you the information, but if one failed and maybe gained, you know, they feel, oh, maybe I failed, uh, when I start to ask about what they eat, they might not want to give me all the information……’

(D2)

Although there appeared to be a gap between the trial dietitians and trial participants in terms of the viewpoints related to the types of information required for dietary intake reporting, all trial dietitians reported that trial participants’ intake reporting improved during the trial, due to trial participants becoming more familiar with the process of diet history interviews.

‘I think they get better at it as they went along, so if they were at 12 months or 9 months they are very good at giving the diet history, because they knew the types of the questions that would be asked, but I remember the first lots of people, that were just doing the initial one really found the process quite hard, and couldn’t remember…… what they usually eat,’

(D3)

## Discussion

The present pilot study implemented mixed methods by using SDVs and in-depth interviews and provided a novel addition to evaluate the accuracy of dietary intake data. The analysis identified that when using diet history interviews, the highest level of discrepancy in the dietary intake data coding process by the trial dietitians occurred during the verification process between the transcripts and nutrition analysis software. Although it is important to interpret these findings with caution, due to the high level of detail of dietary intake data required for subsequent data coding, both the trial dietitians and trial participants played a role in providing incomplete dietary intake information during diet history interviews, consequently influencing the coding process. The issue was suggested to be due to recall bias, as well as the dietitians’ awareness that collected dietary data needed to be obtained in a way that was suitable for entry into the nutrition analysis software. This knowledge appeared to influence the process of interviewing participants and recording their intakes, suggesting that subconscious interpretation during dietary intake data collection was common.

Understanding the point at which discrepancies in dietary data collection and coding may occur is required to improve data quality. The discrepancies observed in the present study are likely to occur during coding of intake quantities. The finding of the present exploratory study is consistent with a previous study described by Gibson et al [20]. In the Airwave Health Monitoring study, the discrepancy rate of portion weight collected by using 7-day food records was 55% [20]. This may be influenced by the high level of detail required for long-term dietary intake data derived from the open-ended methods such as diet history interview and multiple day food records. Difficulties with estimating and recalling quantities of food intake have also been well established in the literature [2, 3, 44]. Portion size estimation is a major concern for determining quantities of food intake. Portion size estimation is determined by perception, conceptualisation and memory [45, 46]. When using portion size estimation aids, perception is the ability to estimate the portion sizes by viewing aids. Conceptualization refers to the ability to form the portion sizes mentally without presenting the actual portion size in front of them. Memory refers to the ability to recall the portion size, which is closely related to conceptualization [46, 47]. Thus, it may indicate that measurement error in the portion size estimation will always present in self-report dietary assessment methods [48].

Knowing which food groups may be more challenging for dietary data collection may help to improve data quality. We found that the food group “vegetable products and dishes” was prone to a discrepancy in reporting quantities and frequencies of intake. This appears to be the result of day-to-day variation in consumption, contributed by the large number of and the seasonal variation in vegetables [49, 50]. The intake of vegetables may be further complicated by use in mixed dishes. During a diet history interview, dietary intake is recalled generally from the first meal of the day through to the end of the day [43]. Mixed dishes are consumed during main meal occasions. Unlike individual foods, mixed dishes are a mixture of individual foods known as ingredients, such as meat, vegetables and/or cereals. The proportions and quantities of the individual foods in mixed dishes vary by participant, which are more likely to be determined by individual consumption preference and food availability in the household, rather than physically measuring the actual quantities [51]. Thus, trial participants might have been unable to report on exact quantities of consumed foods. The literature suggests that supportive tools may be required to be developed and incorporated into the nutrition analysis software to standardise the practice and facilitate a more consistent dietary intake coding process, such as algorithms used to systematically calculate the unknown quantities [9]. In addition, the trial dietitians also suggested that when reporting foods, the trial participants appeared to have little idea on dietary information required to be reported. It may indicate that strategies to improve data quality, such as educating participants on reporting consumption, particularly of mixed dishes may be required to facilitate the subsequent coding process by the trial dietitians.

A wide range of food-based intake data with different levels of detail are collected using diet history interviews. When using a short term dietary assessment method, the 24-hour recall, the mean number of foods per recall ranged from 15 to 37, much less than the number of data collected using the long term methods [15]. As some nutrients are stored in the body, and recommendations of food and nutrient intakes are suggested to be met over time, rather than on daily basis, an approximation of usual dietary intake offers more information for dietary intake than intake on a given day or for a short period. Moreover, intakes of food and beverage from an individual tend to change from day to day. The fluctuations around individual usual mean intake reflect true eating habits in free-living conditions. Thus, usual intake is of interest in most nutrition research. The ability of the diet history method to provide detailed usual dietary intake data, increasing the precision in capturing dietary intake is considered to be a major strength of diet history interview method [7, 43]. However, when coding such usual dietary intake data into nutrition analysis software, the food items and their exact quantities and frequencies are required. In our study, the trial dietitians suggested that during intake recall, the trial participants appeared to experience cognitive difficulties in retrieving and recalling intake information, estimating and judging what they have eaten, influencing their ability to report their detailed intake. Thus, the trial participants were unable to provide adequate information on how much and how often they consumed the food for subsequent data coding in the nutrition analysis software. These results highlight the challenges when collecting and coding detailed dietary intake data, and provide insights into potential reasons for the discrepancies observed in the present study.

Challenges in collecting dietary data and coding it into nutrition analysis software will always occur, whether these arise from recall bias of the trial participants, or a lack of appropriate foods in the food composition database used. The advantage of the diet history interview as a dietary assessment method is that is interviewer administered, which in the case of this study was experienced, research dietitians. Thus, the trial dietitians can clarify and interpret reported dietary intake information during data collection for subsequent data coding. As a result, professional judgement was often used to support the process of dietary data collection and coding. This was observed through the dietitians’ knowledge of the requirements of subsequent data coding to determine the information collected during diet history interviews. The findings in the interviews also revealed that strategies were applied to find the closest substitute when items were not found in nutrition analysis software, such as utilizing food labels and using professional judgment based on the trial participants’ habitual intake. Professional judgement is therefore an important component of dietary data collection and coding, although in the case of items not found in software, detailed protocols on the practice of systematically handling these items may be useful.

Given the recognition of measurement error related to self-report dietary intake data, complete accuracy was not expected; however, there were several limitations of the analyses. There are three components comprising dietary intake data—the food item, its quantity and frequency of consumption, but information on intakes of quantity and frequency is revealed via food item reporting and food quantification [9]. The food item reporting tends to be influenced by recall bias and social desirable reporting behavior [44, 52], which was not addressed in the present analyses. The results of this exploratory study may be subject to bias as the trial participants and trial dietitians involved had already built rapport, compared with those who chose not to participate. The education provided by the intervention arms in clinical trials may also influence dietary intake reporting [53, 54]. Investigator subjectivity may also be involved. The trial participants were overweight or obese, who may be more likely to misreport dietary intake [55]. Furthermore, the sample was also small, so the findings are not generalizable but rather provide insights into the nature of the problem. Although different nutrition analysis software may yield different results, the present study was only meant to explore currently the dietary intake data coding practice in the context of a food-based RCT. Further study is required to provide robust evidence on the dietary intake data coding practices. Notwithstanding this, given the acknowledgement of the limitations of food-based RCTs, advances in the dietary intake data quality in a clinical research setting may also provide insights on dietary intake data derivation process of community-based intervention research and for cohort studies.

In conclusion, accurate dietary intake data is required in clinical settings to provide robust dietary recommendations. In addition to dietary assessment validation studies, the present analyses applied a novel method to examine the dietary intake data coding process at a much deeper level. Applying mixed methods including quantitative (SDV) and qualitative (in-depth interview interviews) assessment methods allowed an exploration of core drivers of quality, subsequently providing recommendations on practice improvement. The findings suggest that although detailed dietary intake data offers better information on food-based intakes, obtaining accurate intakes of quantities and frequencies of foods consumed are challenging due to the inherent limitations of self-reported dietary intake data and the high level of detail required for the dietary intake data coding. The level of detail required is a consideration for the accuracy of dietary assessment. In addition to professional judgement, educating participants on reporting consumption and incorporating supportive tools to deal with unknown intakes of quantities may facilitate a more consistent the dietary intake data coding process and improve data quality.

## Supporting information

S1 Fig Audio-recorder location in the consultation rooms. The arrow and box indicate the location of the audio-recorder in the consultation room.
(TIF)

S1 Table Australian Food, Supplement, and Nutrient Database for Estimation of Population Nutrient Intakes 2007 major food groups and example food items. (DOCX)

S2 Table Outline of questions of semi-structured interview guides. (DOCX)

S3 Table Characteristic of trial participants(n = 20). (DOCX)
