# Supplementary figures and images for: Evaluation of the dietary intake data coding process in a clinical setting: Implications for research practice
Source: PLoS One. 2019 Aug 12;14(8):e0221047. doi: 10.1371/journal.pone.0221047 (PMC6690518; doi:10.1371/journal.pone.0221047)

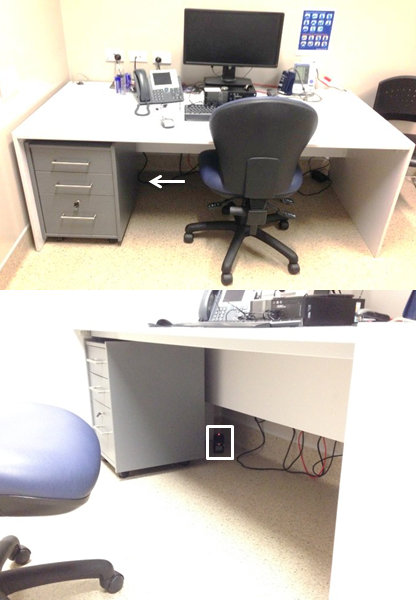

Supplement: S1 Fig — The arrow and box indicate the location of the audio-recorder in the consultation room. (TIF) [file pone.0221047.s001.tif]
