# Supplementary material for: Evaluation of the dietary intake data coding process in a clinical setting: Implications for research practice
Source: PLoS One. 2019 Aug 12;14(8):e0221047. doi: 10.1371/journal.pone.0221047 (PMC6690518; doi:10.1371/journal.pone.0221047)
Supplement: S1 Table — (DOCX) [file pone.0221047.s002.docx]

**S1 Table.** Australian Food, Supplement, and Nutrient Database for Estimation of Population Nutrient Intakes 2007 major food groups and example food items.

| **Food code and group name** | **Examples** |
| --- | --- |
| 11 Non-alcoholic beverages | Tea, coffee, juice, cordial, soft drink, water |
| 12 Cereals and cereal products | Bread, breakfast cereals, noodles, pasta, rice |
| 13 Cereal based products and dishes | Biscuits, cakes, muffins, pastries, pizza, burger, pancakes |
| 14 Fats and oils | Butter, margarine, oils |
| 15 Fish and seafood products and dishes | Fresh fin fish, frozen fin fish, smoked fish, canned fish, prawn, squid, fish cake, tuna mornay with cheese, garlic prawn |
| 16 Fruit products and dishes | Apple, pear, berries, oranges, peaches, banana, melon, dried fruit, apple crumble |
| 17 Egg products and dishes | Eggs, egg dishes such as scrambled eggs, omelette, mousse |
| 18 Meat, poultry and game products and dishes | Beef, veal, lamb, pork, chicken, sausage, bacon, ham, dried meats, crumbled meats, meat bolognaise pasta sauce, casserole, curries |
| 19 Milk products and dishes | Cow milk, yoghurt, cream, cheese, ice cream, rice pudding, cheese cake |
| 20 Dairy substitutes | Soy milk, soy-based yoghurt |
| 21 Soup | Tomato-based soup, vegetable soup, meat-based soup, dry soup mix |
| 22 Seed and nut products and dishes | Pumpkin seeds, linseed, sesame seed, peanuts, peanut butter, almond, coconut milk |
| 23 Savoury sauces and condiments | Gravy, savoury sauces, pickles, salad dressing |
| 24 Vegetable products and dishes | Potatoes, cabbage, carrots, lettuce, beans, fresh herbs, tomato, pumpkin, sweetcorn, onion, salad |
| 25 Legume and pulse products and dishes | Chickpeas, kidney beans, red lentils, dhal (legume curry) |
| 26 Snack foods | Potato crisps, popcorn, corn chips, pretzels |
| 27 Sugar products and dishes | Sugar, honey, jam, |
| 28 Confectionary and cereal/nut/fruit/seed bars | Chocolate, muesli bars, lollies and chewing gum |
| 29 Alcoholic beverages | Beers, wines, sprits, cocktails |
| 30 Special dietary foods | Meal replacement |
| 31 Miscellaneous | Salt, herbs, species, yeast, vegemite, stock |
