# Supplementary material for: Evaluation of the dietary intake data coding process in a clinical setting: Implications for research practice
Source: PLoS One. 2019 Aug 12;14(8):e0221047. doi: 10.1371/journal.pone.0221047 (PMC6690518; doi:10.1371/journal.pone.0221047)
Supplement: S2 Table — (DOCX) [file pone.0221047.s003.docx]

**S2 Table.** Outline of questions of semi-structured interview guides

| **Dietary intake data collection** |
| --- |
| **1.** What do you think are the main barriers or issues that impact on the dietary intake data collection? |
| **2.** How long did you spend with each participant collecting dietary intake data? |
| **3.** In an ideal world, but also balancing participant burden, how long do you think it is suitable to spend with the participant for collecting dietary intake data? |
| **4.** In an ideal world, if you could create any resource possible to assist you when collecting dietary data, what would it look like or what form would it take? |
| **5.** Could you please describe anything you would like to address when you train a dietitian to collect dietary intake data? |
| **6.** What sort of training, if any, do you think you might need to improve your skills to collect dietary data? |
| **7.** Could you please describe anything you would do differently now when you start working as a dietitian to collect dietary data? |
| **Dietary intake data entry** |
| **8.** What do you think are the main barriers or issues that impact on dietary intake data entry? |
| **9.** If you come across food without an exact match in software, which resources do you use to assist you when entering dietary data for that food? |
| **10.** If you could create any resource possible to assist you when entering dietary data, what would it look like and what format would it take? |
| **11.** How long did you spend to enter one collected dietary intake data record? |
| **12.** If you could create any resource possible to assist you to save time when entering dietary data, what would it look like and what format would it take? |
| **13.** Could you please describe anything you would like address when you train a dietitian to enter dietary intake data? |
| **14.** What sort of training, if any, do you think you might need to improve your skills in entering dietary data? |
| **15.** Could you please describe anything you would do differently now when you start working as a dietitian to enter dietary data? |
