# Supplementary material for: Evaluation of the dietary intake data coding process in a clinical setting: Implications for research practice
Source: PLoS One. 2019 Aug 12;14(8):e0221047. doi: 10.1371/journal.pone.0221047 (PMC6690518; doi:10.1371/journal.pone.0221047)
Supplement: S3 Table — (DOCX) [file pone.0221047.s004.docx]

**S3 Table. Characteristic of trial participants(n=20)**

| **Characteristic** |  | **P value** |
| --- | --- | --- |
| Gender (%) |  |  |
| Male^1^ | 9 (45) |  |
| Female^1^ | 11(55) |  |
| Age (year)^1^ | 44.75±6.81 |  |
| Weight (kg) |  | P=0.045 |
| At baseline^1^ | 89.84±16.64 |  |
| 12 month^1^ | 87.14±15.64 |  |
| Weight change (kg)^2^ | -1.45 (-4.38 - 0.85) |  |
| BMI (kg/m^2^) |  | P=0.035 |
| At baseline^1^ | 30.44±4.36 |  |
| 12 month^1^ | 29.53±3.86 |  |
| BMI change (kg/m^2^)^2^ | -0.48 (-1.48 - 0.24) |  |
| Waist circumference (cm) |  | P=0.002 |
| At baseline^1^ | 98.65±12.43 |  |
| 12 month^1^ | 93.45±13.78 |  |
| Waist circumference change (cm)^2^ | -4.75 (- 8.50 – 1.00) |  |

^1^ Mean ± Standard Deviation

^2^ Median (Interquartile range)
